# Pigmentary colouration of hairy carpenter bees, genus *Xylocopa*

**DOI:** 10.1007/s00114-023-01854-9

**Published:** 2023-05-23

**Authors:** Doekele G. Stavenga

**Affiliations:** Groningen Institute for Evolutionary Life Sciences, University of Groningen, NL-9747 AG Groningen, The Netherlands

**Keywords:** Bile pigment, Reflectance spectrum, Granule scattering, Spectral sensitivity

## Abstract

Carpenter bees can display distinct colouration patterns due to structural coloured wings and/or coloured hairs on their bodies. Females of the sexually dichromatic *Xylocopa caerulea* are marked by strongly blue-pigmented hairs on the head, thorax and abdomen. The thorax of female *X. confusa* is covered by yellow-pigmented hairs. The diffuse pigmentary colouration of the blue and yellow hairs is effectively enhanced by strongly scattering granules. The absorption spectrum of the blue pigment of *X. caerulea* has a maximum at 605 nm and is probably a bilin (a bile pigment). The absorption spectrum of the yellow pigment of *X. confusa* has a maximum at 445 nm and may be a pterin. The thoracic hairs of female *X. confusa* contain also a minor amount of the bilin. The reflectance spectra of the pigmented hairs suggest that the pigments are tuned to the spectral sensitivity of the bees’ photoreceptors and provide spectral contrast with a green background.

## Introduction

Carpenter bees are species in the extensive genus *Xylocopa*, well known for their nesting behaviour in hard plant material (Gerling et al. 1989). Many species have brightly coloured bodies and/or wings, and sexual dichromatism is widespread among *Xylocopa* species (Gerling et al. 1989; Blaimer et al. 2018). Females of most species are conspicuous with their black or blue colouration, which may be variegated with lighter-coloured pubescence. Males either resemble females or may be completely covered with a light brown, light green or yellowish green pubescence (Gerling et al. 1989). The distinct body colouration of the bees presumably functions in intraspecific recognition. For instance, studies of mate detection in the male carpenter bee *Xylocopa tenuiscapa* have demonstrated a clear role of vision (Somanathan et al. 2009; Somanathan et al. 2017), suggesting that the body colouration plays an important role.

A specific case of interest is that of *Xylocopa iris*, which has distinctly blue-reflecting wings, similarly as the structurally coloured wings of the related carpenter bee *X. latipes* (Stavenga et al. 2023). The body pattern of a resting female *X. iris* with her metallic reflecting wings is mimicked by the orchids *Ophrys spuneri* and *O. sipontensis* (Spaethe et al. 2007). Males are attracted by the orchids, and via their subsequent visits, they function in pollinating the orchids. Whereas the female *X. iris* has wings displaying an ultraviolet-blue structural colouration, females of the related species *X. caerulea* exhibit a blue-coloured thorax, densily covered with blue-pigmented hairs (Mason 1926; Mawdsley 2016). *X. confusa* females practice a similar method of pigmentary colouration, but their hairs are yellow.

Here I investigate the colouration of these exemplary *Xylocopa* species, applying (micro)spectrophotometry, to gain further understanding of carpenter bee pigmentary vs structural colouration.

## Materials and methods

**Fig. 1:**
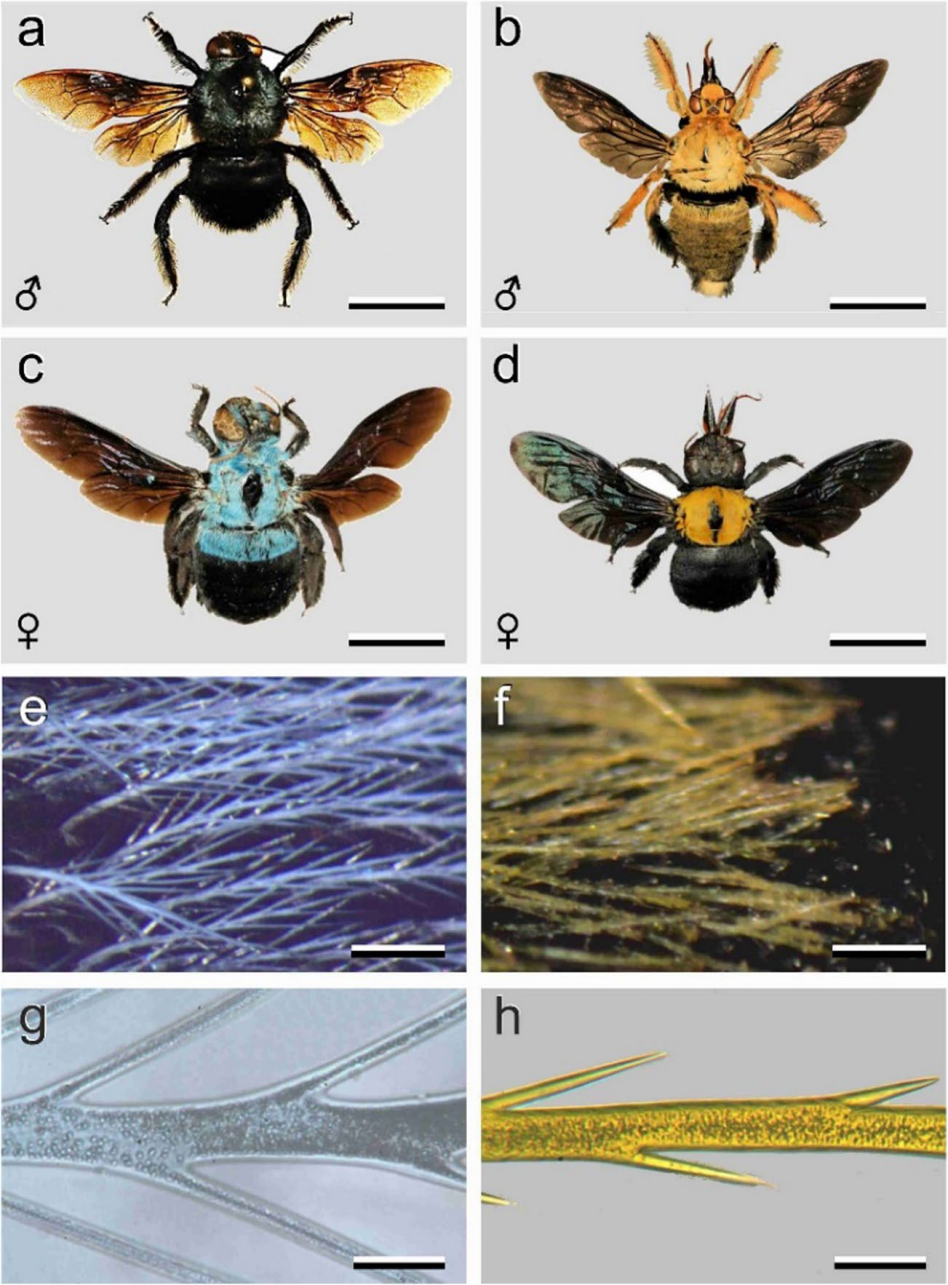
Hairy carpenter bees. **a**, **c** *X. caerulea*. **b**, **d** *X. confusa*, **a**, **b** Males; **c**, **d** Females. **e**, **f** Epi-illumination micrographs of the thorax of female *X. caerulea* and *X. confusa*. **g** A thoracic hair of a female *X. caerulea* immersed in oil (epi-illumination, dark field), **h** A thoracic hair of a female *X. confusa* immersed in oil (transmitted light). Scale bars: **a**-**d** — 1 cm; **e**, **f —** 0.2 mm; **g**, **h** — 25 μm

Specimens of *X. caerulea* and *X. confusa* were obtained from a commercial source (thebugmaniac.com, presently etsy.com). The specimens were photographed with a Canon EOS digital camera (Fig. 1b–d; Fig. 1a is modified Fig. 5 of Mawdsley 2016). Small thorax areas (Fig. 1e,f) were photographed with an SZX16 stereomicroscope (Olympus, Tokyo, Japan) and hairs embedded in immersion oil (refractive index 1.515) (Fig. 1g,h) with a Zeiss Universal microscope (Zeiss, Oberkochen, Germany). Reflectance spectra of local thorax areas (~1 mm) of *X. caerulea* and *X. confusa* were measured with a bifurcated probe, directed about normally to the body surface, connected to a halogen-deuterium lamp (AvaLight-D(H)-S) and an Avantes AvaSpec-2048-2 CCD detector array spectrometer (Avantes, Apeldoorn, Netherlands). The reference for the reflectance measurements was a white diffuse standard (Avantes WS-2). Absorbance spectra of the pigments in the thoracic hairs were derived from transmittance measurements of oil-immersed hairs with a microspectrophotometer (MSP), consisting of a Leitz Ortholux microscope with a LUCPlanFL N 20x/0.45 objective (Olympus, Tokyo, Japan) and the detector array spectrometer. The light source was a 150 W Xe-lamp (Osram). Rhodopsin spectra were calculated using a visual pigment template (Stavenga 2010).

## Results

Carpenter bees vary widely in their colouration pattern (Fig. 1). Male and female *X. caerulea* as well as *X. confusa* strongly differ in appearance. The male *X. caerulea* body features a blackish cuticle (Fig. 1a), while the male *X. confusa* has faint-yellowish hairs all over the body (Fig. 1b). The female *X. caerulea* has a bright cover of blue hairs on both the thorax and part of the abdomen and head (Fig. 1c), while the female *X. confusa* has bright yellow hairs exclusively on the thorax (Fig. 1d). The wings of *X. caerulea* and *X. confusa* are light- to dark-brown with at most a slight metallic shimmer (Fig. 1a–d).

The coloured hairs of *X. caerulea* and *X. confusa* display a diffuse colouration, which has obviously a pigmentary basis (Mason 1926). Micrographs show this into more detail. The branched hairs have a cross-section of the order of 20–30 μm (Fig. 1e–h). A thin cortex envelopes the pigmented hair volume, which contains numerous micrometer-sized granules (Fig. 1g, h). The latter function as effective diffusers for the pigmentary colouration of the hairs.

**Fig. 2:**
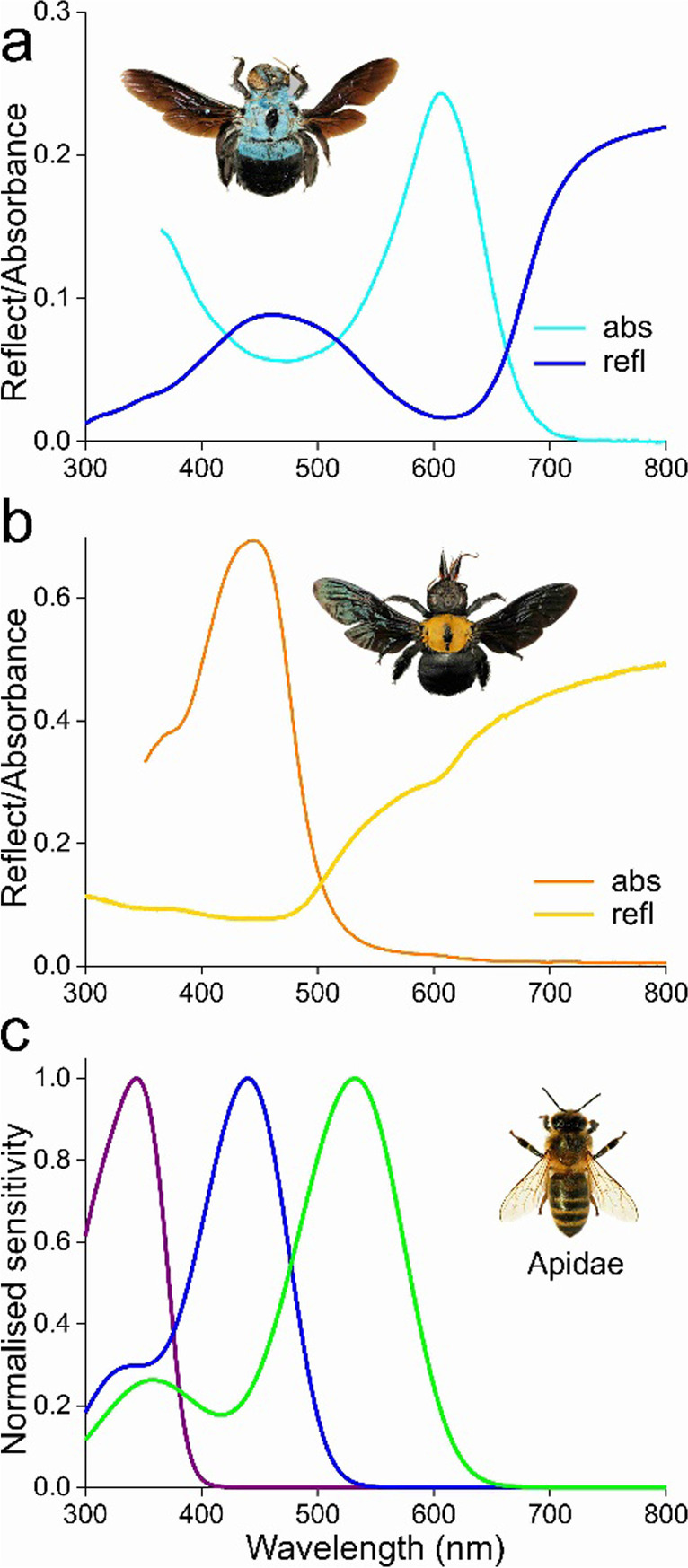
Spectral characteristics of carpenter bees and bee photoreceptors. **a** Reflectance (refl) spectrum of the thorax of *X. caerulea* measured with a bifurcated probe, and absorbance (abs) spectrum derived from transmittance microspectrophotometry on a thoracic hair immersed in oil. **b** As **a** for *X. confusa*. **c** Sensitivity spectra of bee photoreceptors due to rhodopsins with absorption spectra peaking at 346, 440 and 532 nm, indicative for Apidae photoreceptors

To characterize the pigments in the carpenter bees’ bodily hairs, reflectance spectra were measured using a bifurcated probe (Fig. 2a, b). The reflectance spectrum of the thorax of female *X. caerulea* has a broad band in the blue wavelength range, with a trough around 600 nm, indicating the presence of a pigment strongly reducing the reflection in the red wavelength range (refl, Fig. 2a). Indeed, transmittance microspectrophotometry on hairs isolated and immersed in immersion oil yielded an absorbance spectrum with a distinct band peaking at 605 nm (abs, Fig. 2a).

The same measurements executed on female *X. confusa* resulted in an absorbance band peaking at 445 nm, demonstrating the presence of a strongly blue absorbing pigment, which causes the yellow colour of the hairs (Fig. 2b). Interestingly, the absorbance spectrum shows a very minor hump at ~605 nm, which has a counterpart in the thorax reflectance spectrum measured with the bifurcated probe. The small kink around 605 nm suggests that *X. confusa* hairs contain a minor fraction of *X. caerulea*’s blue pigment.

## Discussion

The colouration of several hymenopterans, specifically bumblebees, is created by a cover of pigmented hairs. Already a century ago, Mason concluded that the blue colour of the thoracic hairs of *X. caerulea* is pigmentary (Mason 1926). Of the species included in the *Xylocopa* group *Cyaneoderes*, five have females with bright blue pubescence. These species form a well-defined and (due to its rare blue colouration) an easily recognised group within the *Xylocopa* subgenus *Koptortosoma* (Mawdsley 2016). The pigment responsible for the blue colour is unknown, but the shape of its absorption spectrum indicates that it is a bilin, the class of bile pigments that is expressed in various insect larvae (Junge 1941). Bilins are also found in some adult insects, but they are then mostly expressed jointly with carotenoids, so to achieve camouflage against a green background (Fuzeau-Braesch 1972; Choussy and Barbier 1973; Stavenga et al. 2010). Nevertheless, blue pigmentary colouration is rather unique among insects.

The yellow colour of the hairs of *X. confusa* is very common among bees. The carpenter bee *X. appendiculata*, pollinating exclusively the orchid *Calanthe striata* (Sugiura 2013), has a cover of yellow hairs, similar to female *X. confusa*. Also, many bumblebees have yellow hairs on their body, but the underlying yellow pigment has not yet been identified. It is not an ommochrome or carotenoid and may be a pterin (Rapti et al. 2014; Pimsler et al. 2017).

Structural coloured wings of carpenter bees can play a dominant role in their display pattern. Nanosized structures, most likely multilayers in the strongly melanized wings as in *X. latipes*, cause the distinct blue-violet colour of for instance *X. valga* and *X. iris*. The short-wavelength reflections presumably have a function in intraspecific communication, as is indicated by orchids that mimic the females (Spaethe et al. 2007). Blue structural colouration is encountered also in other hymenopterans, e.g., the blue-banded bee *Amegilla cingulate* and the neon cuckoo bee *Thereus nitidulus* (Saranathan et al. 2015). However, here in both male and female, the photonic structure consists of a hexagonal array of air-filled tubules in the chitinous scales (Fung 2005).

The spectral characteristics of the blue wing reflections and of the blue or yellow hairs suggest a distinct visual function, that is, the colours seem to be tuned to the spectral sensitivity of the bee’s photoreceptors. Sensitivity spectra of carpenter bee photoreceptors have not yet been reported, but the spectra of the various photoreceptor types of hymenopterans appear to be generally quite well-defined (Peitsch et al. 1992). The peak wavelengths of the studied ultraviolet (UV), blue (B), and green (G) receptors only slightly scatter. Figure 2c shows the absorption spectra of UV, B, and G rhodopsins peaking at 346, 440, and 532 nm, respectively, being the averages of the known bee photoreceptors (Van der Kooi et al. 2021). Comparing the reflectance spectra measured of wings and hairs with these photoreceptor spectra suggests that the blue wings and hairs are mainly tuned to the B-receptors, while the yellow hairs predominantly activate the G-receptors (Fig. 2). The high reflectance at the longer wavelengths (> 650 nm) is irrelevant for bee vision, as the spectral sensitivity of the photoreceptors is there negligible. The blue and yellow colouration will create a distinct spectral contrast of the carpenter bees with the generally green background of plants.
